# One-year mortality after hip fracture surgery: urban–rural differences in the Colombian Andes

**DOI:** 10.1007/s11657-022-01150-5

**Published:** 2022-08-09

**Authors:** Juan-Daniel Duque-Sánchez, Luis-Ángel Toro, Fernando-Iván González-Gómez, Sandra-Milena Botero-Baena, Gustavo Duque, Fernando Gómez

**Affiliations:** 1 Internal Medicine Section, Faculty of Health Sciences, Universidad de Caldas, Manizales, Colombia; 2 Research Group On Geriatrics and Gerontology, Faculty of Health Sciences, Universidad de Caldas, Manizales, Colombia; 3 Department of Medicine-Western Health, Melbourne Medical School, University of Melbourne, St Albans, VIC, Australia; 4 Australian Institute for Musculoskeletal Science (AIMSS), The University of Melbourne and Western Health, St Albans, VIC, Australia

**Keywords:** Hip fracture, Mortality, Rural, Risk factors, Colombia

## Abstract

**Summary:**

To determine urban–rural differences influencing mortality in patients with hip fracture in Colombian Andes Mountains over a 1-year period.

**Purpose:**

To identify the urban–rural differences of sociodemographic variables, fracture-related characteristics, and preoperative and postoperative clinical factors associated with 1-year mortality in patients over 60 years old who underwent hip fracture surgery in the Andes Mountains.

**Methods:**

A total of 126 patients with a fragility hip fracture during 2019–2020 were admitted to a tertiary care hospital. They were evaluated preoperatively and followed up until discharge. Those who survived were contacted by telephone at 1, 3, and 12 months. Univariate, bivariate, and Kaplan–Meier analyses with survival curves were performed. Relative risk was calculated with a 95% confidence interval.

**Results:**

A total of 32.5% of the patients died within 1 year after surgery, with a significant difference between those who resided in rural areas (43.1%) and those who resided in urban areas (23.5%) (RR 1.70; 95% CI, 1.03 to 2.80, *p* = 0.036). In the multivariate analysis, anemia (hemoglobin level ≤ 9.0 g/dL during hospitalization) (RR 6.61; 95% CI, 1.49–29.37, *p* = 0.003), a blood transfusion requirement (RR 1.47; 95% CI, 1.07 to 2.01, *p* = 0.015), the type of fracture (subtrochanteric fracture (RR = 4.9, 95% CI = 1.418–16.943, *p* = 0.005)), and postoperative acute decompensation of chronic disease (RR 1.60; 95% CI, 1.01 to 2.53, *p* = 0.043) were found to be independent predictive factors of 1-year mortality after surgery.

**Conclusions:**

There was a difference in 1-year mortality between patients from rural and urban areas. More studies must be conducted to determine whether rurality behaves as an independent risk factor or is related to other variables, such as the burden of comorbidities and in-hospital complications.

## Introduction

Fragility hip fracture is considered a severe consequence of osteoporosis due to its complications, which include chronic pain, disability, deterioration in quality of life, and premature death [1]. Due to the change in population dynamics, with a worldwide increase in life expectancy and the consequent increase in the number of older adults, growth in the incidence of hip fracture is expected, which is projected to increase from 1.66 million in 1990 to 6.26 million by 2050 [2]. This pathology is directly related to sex and age, occurring more frequently in women and those over 60 years of age [1]. Since the 1990s, epidemiological studies have found a high mortality rate, which can exceed 10% in the perioperative period and the first 30 days [3] and can fluctuate between 18 and 31% in the first year after the event, depending on the population under study and despite proper handling [4]. Early surgical intervention is the variable that has the most apparent impact on mortality in-hospital management [5], but mortality in the first year for older men and women with fragility hip fracture has been reported to be 3–4 times higher than that expected in the general population and to be higher in older men than in older women [6].

Geographic trends of the incidence of fragility hip fractures have been reported, with the highest rates of hip fracture found in Scandinavia and the lowest rates found in Africa [4]. In Latin America, the hip fracture rates are similar to the values reported in North America [4], while urban–rural differences in hip fracture prevalence are well documented, with higher rates in urban areas [7]. However, urban–rural differences in hip fracture mortality are more confusing, with some studies showing no increase [8], others finding higher mortality in rural areas [9, 10], and others finding higher mortality in urban areas [11]. Several explanations have been proposed for these contradictory findings: differences in general health in urban–rural populations, including multimorbidity, could be an important reason [12], and other factors, such as demographics and socioeconomic, social, and environmental factors, could contribute to this difference [11, 13], as well as inequalities between urban and rural municipalities regarding follow-up health care services or rehabilitation services [11].

In Colombia, epidemiological and follow-up studies on the subject are scarce and restricted to only urban areas. One study characterized patients older than 60 years with fragility hip fractures who consulted with a university hospital and followed them up to 6 months, finding mortality of 19% but with a loss of 25.5% of the patients [14]. Another group found a significant difference in mortality at 1 year, decreasing from 20 to 11%, by implementing an orthogeriatric model of care [15]. Furthermore, 478 patients who underwent hip surgery and were treated at a tertiary hospital in Bogotá showed a mortality of 31.6% at the 1-year follow-up. Factors such as multimorbidity, an age > 80 years old, a surgical delay > 4 days, and a hemoglobin level < 10 g/dL were associated with increased 1-year mortality [16]. However, to our knowledge, no study has investigated the relationship between the hip fracture mortality rate in developing countries and urban and rural settings.

The aim of this study was to analyze (1) any urban–rural differences in 1-year mortality and (2) whether possible urban–rural differences in 1-year mortality could be explained by preoperative and postoperative factors in patients older than 60 years undergoing hip fracture surgery in the Andes Mountains.

## Materials and methods

We conducted a 12-month follow-up evaluation of patients with fragility hip fractures admitted to the geriatric-orthopedic ward of a tertiary teaching hospital in Manizales, located in the Colombian Andes Mountains region, between May 2019 and April 2020. Informed consent was obtained from all patients, and the local ethics committee of the hospital approved the study. The inclusion criteria were older patients (60 years and older) who were previously ambulatory and had at least a 1-year postoperative follow-up if they survived. Patients with pathological fractures who underwent resection arthroplasty (Girdlestone), patients who did not undergo surgery, or patients who did not sign the informed consent form were excluded.

The definition delimiting rural was adapted from the recommended Organization for Economic Co-operation and Development (OECD) taxonomy, which defines 3 regions: predominantly urban, intermediate, and predominantly rural. Regions are classified as either rural or urban using a population density threshold [17]. According to the official definition, 23% of the Colombian population lives in rural areas. This study was approved by our Institutional Review Board.

### Variables

For patients who met the inclusion criteria, the field investigators collected the sociodemographic data, fracture-related characteristics, and preoperative and postoperative clinical variables from medical charts and caregivers. The sociodemographic variables were age, sex, health care affiliation (contributive scheme for formal workers and subsidized scheme for those without the ability to pay), and marital status. The fracture-related variables included the type of fracture (extracapsular fractures such as intertrochanteric and subtrochanteric fractures; intracapsular fractures such as femoral neck fractures and periprosthetic fractures), with distinctions between the four types of fractures made through X-rays by orthopedic surgeons, the time from the fall to surgery, and the time from admission to surgery. Concerning the preoperative clinical variables, comorbidity was measured by the total number of self-reported physician-diagnosed chronic conditions (hypertension, diabetes, hyperlipidemia, osteoarthritis, hypothyroidism, cognitive impairment, osteoporosis, lung diseases, psychiatric diseases, chronic kidney disease, and stroke). The Charlson comorbidity index score was determined. Other preoperative variables obtained were the Functional Ambulation Classification (FAC) gait scale score, functional class (NYHA), ASA classification, nutritional status (according to body mass index [BMI]), and screening for cognitive impairment by the Mini–Mental State Examination (MMSE) and Barthel scale. The laboratory preoperative clinical variables obtained were hemoglobin, calcium (normal value 8.5–10.1 mg/dL, corrected by albumin), phosphorus (normal value 2.5–4.5 mg/dL), 25-hydroxyvitamin D3 (deficiency < 20 ng/mL, insufficiency 21–29 ng/mL), parathormone (normal value 14.5–128 pg/mL), and albumin (normal value 3.5–5 g/dL) levels.

Medical and surgical complications during admission and postoperatively were defined in advance based on well-established criteria [18]. Acute decompensation of chronic disease was considered as COPD exacerbation, acute decompensation of heart failure, acute renal failure over preexisting chronic kidney failure, a hyperosmolar state in diabetic patients, and hypertensive emergencies. All acute decompensation events were defined by clinical diagnosis by the practitioner caring for the patient. Other postoperative complications (myocardial infarction, new arrhythmia, deep venous thrombosis, pulmonary embolism, delirium, surgical site infection, skin and soft tissue infection, postoperative urinary tract infection, gastrointestinal hemorrhage, delirium, and the requirement for a blood transfusion) were recorded after a detailed review of the inpatient chart. Surgical and medical complications were recorded during the stay, in addition to mortality during hospitalization and at 1, 3, and 12 months.

### Statistical analysis

Descriptive analysis was carried out by calculating the frequencies and percentages for categorical variables, and means and medians were used where appropriate to describe the data. We performed univariate and multivariate analyses to ascertain the independent risk factors for 1-year mortality. The bivariate analysis was carried out as follows: for qualitative variables, the chi-square test with Yates or Mann–Whitney correction was used, as appropriate, and for continuous variables, according to the distribution of the variable, the independent Student’s *t*-test or the nonparametric Mann–Whitney test was used. Relative risk (RR) was calculated with a 95% confidence interval. The chi-square test was also used to test the relationship between the independent variables and 1-year mortality (dependent variable). Mortality in hip fracture patients and controls was analyzed using Kaplan–Meier survival curves. Statistical analyses were performed using SPSS software, version 25.0 for macOS (SPSS, Chicago, IL, USA). A *p*-value ≤ 0.05 was considered significant.

## Results

Over the study period, 132 patients admitted with a diagnosis of hip fracture were screened, and 126 patients met the inclusion criteria. The planned 1-year follow-up was reached for 124 patients. Patients were between 60 and 99 years old, with an average age of 81. Table 1 summarizes the sociodemographic variables, fracture-related characteristics, and pre- and post-operative clinical variables of the sample according to rural or urban residence. The majority of the patients lived in urban areas (72.4% of the men and 55.6% of the women), and the mean age at hip fracture was slightly lower in urban than in rural municipalities (81 vs. 82 years), with no statistically significant differences. The most common type of fracture was extracapsular fracture (intertrochanteric fracture in 71.4% of the sample) followed by intracapsular fracture (basicervical fracture in 16.6% of the sample), with urban/rural differences. No differences between urban–rural mean time from the fall to surgery were found (6 vs. 7 days) or between the mean time from admission to surgery (4 vs. 5 days). A median Charlson comorbidity index score of 4 was found, and hypertension was the most prevalent chronic disease (66%), followed by diabetes mellitus (30%), dyslipidemia (24%), osteoarthrosis (24.6%), and cognitive impairment (17.5%). No urban/rural statistically significant differences were found. No statistically significant differences emerged when comparing preoperative characteristics, including functional and nutritional aspects, according to urban/rural residence. However, rural patients reported higher dependent functional status than urban patients (25.4% vs. 18.6%, respectively, *p* = 0.167) and had higher ASA scores (score 4–5: 49% vs. 45%, respectively, *p* = 0.75). Furthermore, rural patients reported a lower average Hb level on admission (11.3 g/dL vs. 12.1 g/dL, respectively, *p* = 0.03). The majority of the patients had vitamin D deficiency (mean: 19.7 ng/mL (4–78.7) with lower levels in urban than in rural patients (18.4 ng/mL vs. 21.9 ng/mL). The groups had no significant difference in the serum calcium, phosphorus, parathormone, or albumin levels.

**Table 1:** Characteristics of the cohort according to rural or urban residence

Characteristics	Total (*n* = 126)	Residence		*p*-value
		Rural (*n* = 51)	Urban (*n* = 75)	
Sociodemographics				
Age, median (range), years	81 (60–99)	82 (65–95)	81 (60–99)	0.381
Sex, No. (%)				
Female	97 (77)	43 (84.3)	54 (72)	0.107
Male	29 (23)	8 (15.7)	21 (28)	
Health care affiliation, No. (%)				
Subsidized	68 (54)	32 (62.7)	36 (48)	0.211
Contributive	57 (45.2)	19 (37.3)	38 (50.7)	
Marital status, No. (%)				
Married	66 (52.4)	28 (54.9)	38 (50.7)	0.743
Widowed	37 (29.3)	16 (31.4)	21 (28.0)	
Single	19 (15.1)	6 (11.8)	13 (17.3)	
Fracture-related				
Type of fracture, No. (%)				
Extracapsular	103 (81.7)	44 (86.2)	59 (78.6)	0.033
Intracapsular	21 (16.6)	6 (11.7)	15 (20)	
Periprosthetic	2 (1.6)	1 (2.0)	1 (1.3)	
Time from the fall to surgery, mean (range), hours	96 (24–408)	168 (48–456)	144 (24–1008)	0.082
Time from admission to surgery, mean (range), hours	96 (24–408)	120 (48–360)	96 (24–408)	0.101
Preoperative clinical				
Comorbidity score (Charlson Index), mean (range)	4 (2–12)	4 (2–11)	4 (2–12)	0.431
Comorbidities, No. (%)				
Hypertension	84 (66.7)	38 (74.5)	46 (61.3)	0.124
Diabetes	38 (30.2)	15 (29.4)	23 (30.7)	0.880
Hyperlipidemia	31 (24.6)	15 (29.4)	16 (21.3)	0.301
Osteoarthritis	31 (24.6)	13 (25.5)	18 (24)	0.849
Hypothyroidism	22 (17.5)	6 (11.8)	16 (21.3)	0.165
Cognitive impairment	22 (17.5)	11 (21.6)	11 (14.7)	0.316
Osteoporosis	22 (17.5)	8 (15.7)	14 (18.7)	0.665
Chronic obstructive pulmonary disease	19 (15.1)	6 (11.8)	13 (17.3)	0.391
Psychiatric disease	18 (14.3)	6 (11.8)	12 (16)	0.505
Chronic kidney disease	16 (12.7)	7 (13.7)	9 (12)	0.775
Functional Ambulation Classification, No. (%)				
Independent (4–5)	99 (78)	38 (74.5)	61 (81.3)	0.167
Dependent (0–3)	27 (23)	13 (25.4)	14 (18.6)	
Functional Class (NYHA), No. (%)				
I	41 (32.5)	13 (25.5)	28 (37.3)	0.316
II	74 (58.7)	34 (66.7)	40 (53.3)	
III	11 (8.7)	4 (7.8)	7 (9.3)	
ASA Physical Status Classification, No. (%)				
I	3 (2.4)	2 (3.9)	1 (1.3)	0.755
II	64 (50.8)	24 (47.1)	40 (53.3)	
III	54 (42.9)	23 (45.1)	31 (41.3)	
IV	5 (4)	2 (3.9)	3 (4.0)	
Nutritional status (BMI), No. (%)				
Underweight	22 (17.5)	9 (17.6)	13 (17.3)	0.828
Normal range	63 (50)	25 (49.0)	38 (50.7)	
Overweight	10 (7.9)	3 (5.9)	7 (9.3)	
Obesity	31 (24.6)	14 (27.5)	17 (22.7)	
Mini–Mental State Examination, No. (%)				
Positive for cognitive impairment	61 (48.4)	29 (56.9)	32 (42.7)	0.118
Negative for cognitive impairment	65 (51.6)	22 (43.1)	43 (57.3)	
Barthel index, median (range)	100 (5–100)	100 (10–100)	100 (5–100)	0.386
Laboratory variables				
Hemoglobin, mean (SD), g/dL	11.8 (1.95)	11.3 (1.9)	12.1 (1.9)	0.033
Calcium, mean (SD), mg/dL	8.6 (0.2)	8.2 (0.6)	8.2 (0.6)	0.873
Phosphorus, mean (SD), mg/dL	3.66 (0.76)	3.5 (0.8)	3.7 (0.7)	0.220
Albumin, mean (SD), g/dL	3.46 (0.46)	3.4 (0.5)	3.5 (0.4)	0.076
25-Hydroxyvitamin D3, mean (range), ng/mL	19.7 (4–78.7)	21.9 (4–78.7)	18.4 (4–77)	0.417
Parathormone, mean (range), pg/mL)	57.56 (12.6–819)	59.5 (12.6–819)	56.5 (14.2–207.1)	0.566
Postoperative clinical				
Postoperative complications, No. (%) (30-day)				
Requirement for blood transfusion	68 (54)	34 (66.7)	34 (45.3)	0.018
Acute decompensation of chronic disease	46 (36.2)	24 (47.1)	22 (29.3)	0.043
Delirium	34 (26.8)	18 (35.3)	16 (21.3)	0.083
Urinary tract infection (UTI)	16 (12.6)	10 (19.6)	6 (8)	0.055
Pneumonia	9 (7.1)	6 (11.8)	3 (4)	0.191
Gastrointestinal hemorrhage	7 (5.5)	5 (9.8)	2 (2.7)	0.187
Myocardial infarction or cardiac arrest	5 (3.9)	3 (5.9)	2 (2.7)	0.658
Deep vein thrombosis (DVT)	4 (3.2)	3 (5.9)	1 (1.3)	0.316
Skin and soft tissue infection (SSI)	3 (2.4)	0 (0)	3 (4)	0.395
Mechanical complications	3 (2.4)	2 (3.9)	1 (1.3)	0.734
Surgical site infection	2 (1.6)	1 (2)	1 (1.3)	1.000

With regard to the postoperative patient characteristics, the most frequent in-hospital complications were the requirement for a blood transfusion (54%), acute decompensation of chronic disease (36.2%), delirium (26.8%), and urinary tract infection (12.6%). A statistically significant difference was found between urban and rural patients for different variables. In particular, patients from rural areas had more complications, such as requiring a blood transfusion, and higher percentages of acute decompensation of chronic diseases.

The multivariate analysis allowed us to identify a total of four independent predictive factors (Table 2). Extracapsular fracture (RR = 4.9, 95% CI = 1.418–16.943), anemia (an Hg level ≤ 9.0 g/dL during hospitalization) (RR = 6.61, 95% CI = 1.491–29.372), postoperative acute decompensation of chronic disease (RR = 1.60, 95% CI = 1.017–2.532), and the requirement for a blood transfusion (RR = 1.47, 95% CI = 1.073–2.016) were found to be statistically significant factors associated with 1-year mortality after surgery among rural patients.

**Table 2:** Factors related to rural residence and their RRs

Characteristic	Risk ratio	95% CI	*p*-value
Hemoglobin level ≤ 9 g/dL	6.61	(1.491–29.372)	0.003
Subtrochanteric fracture	4.90	(1.418–16.943)	0.005
Postoperative acute decompensation of chronic disease	1.60	(1.017–2.532)	0.043
Blood transfusion requirement	1.47	(1.073–2.016)	0.018

At the 1-year follow-up after surgery, 41 patients (32.5%) had died; when comparing the occurrence of death between patients in urban and rural areas, statistically significant differences were found (Table 3). In-hospital mortality reached 12.7%, 1-month mortality reached 20.6%, and 3-month mortality reached 26.2%; the mortality rates were higher for rural patients than for urban patients for in-hospital, 30-day, and 3-month mortality, with no significant differences.

**Table 3:** Mortality according to residence

Mortality, No. (%)	Total	Rural	Urban	*p*-value
In-hospital	16 (12.7)	10 (19.6)	6 (8)	0.055
1 month	26 (20.6)	14 (27.5)	12 (16)	0.119
3 months	33 (26.2)	18 (35.3)	15 (20)	0.055
12 months	41 (32.5)	22 (43.1)	19 (25.3)	0.036

**Fig. 1:**
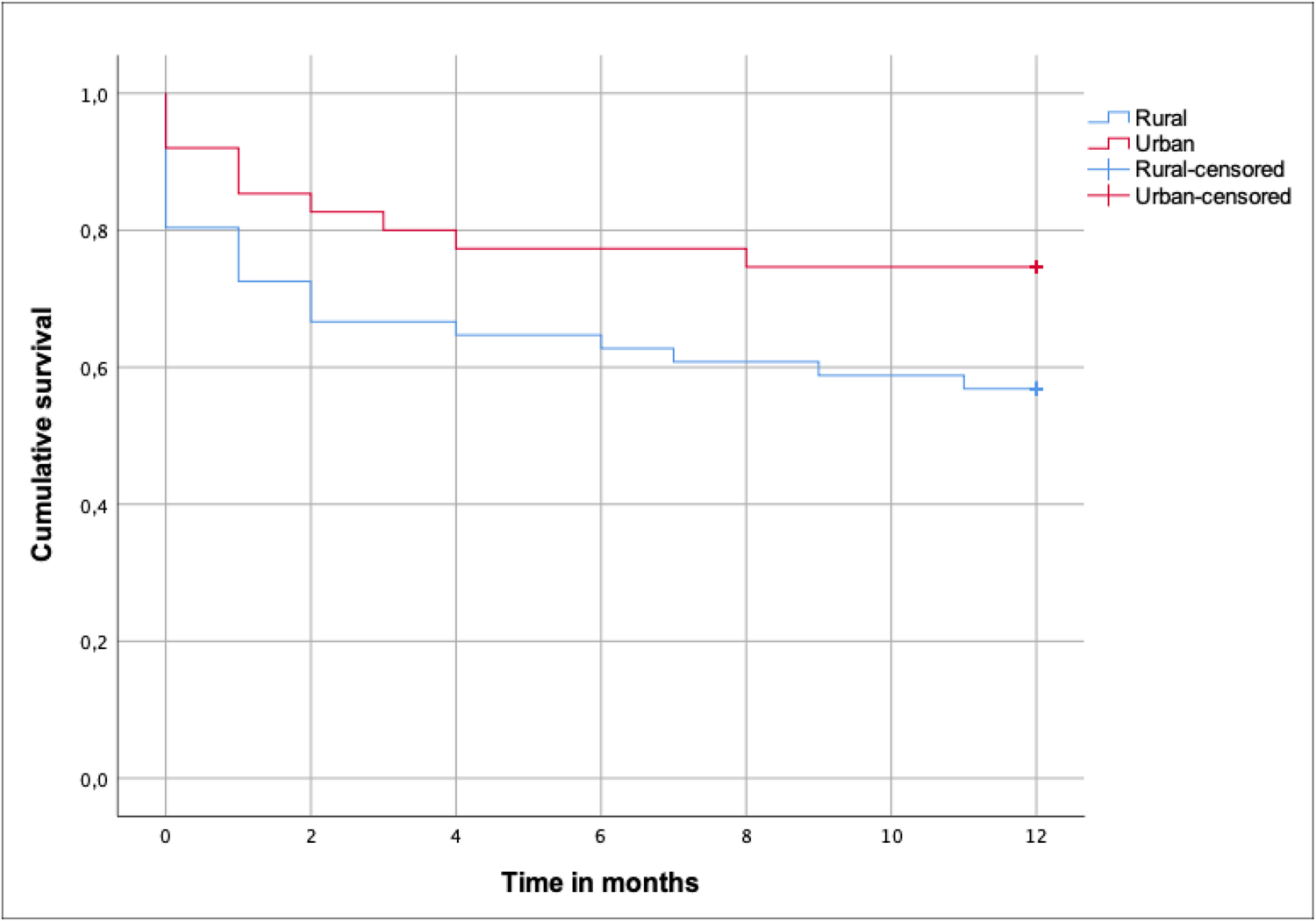
The Kaplan–Meier survival curve shows 1-year mortality after fracture among rural and urban older people

Significant differences in mortality rates were seen using the log-rank statistical analysis between rural and urban areas (63.5% versus [vs.] 57.0%, respectively, *p* = 0.07), but were not seen for males (74.5% vs. 67.4%, *p* = 0.13) or females (59.1% vs. 52.9%, *p* = 0.20). The 1-year standardized mortality rate was 341.3 (95% CI, 162.5–520.1) for rural patients and 301.6 (95% CI, 212.4–391.8) for urban patients. This implies that mortality rates in this patient population were 3.4 times higher for rural areas and 3.0 times higher for urban areas than those expected in the general population during this 1-year period. Cumulative survival for fragility hip fracture patients in the rural patient group was lower than that for patients in the urban group (Fig. 1). A statistically significant difference was seen between rural and urban patients in the log-rank statistical analysis (*p*, 0.032) and Breslow analysis (*p*, 0.029).

## Discussion

In our population-based hip fracture study, rural patients suffered exceptionally high mortality at 1-year follow-up after the event compared to the urban group (43% vs. 25%, respectively). Anemia (a Hb level ≤ 9.0 g/dL during hospitalization), a blood transfusion requirement, the type of fracture (extracapsular fracture), and acute postoperative decompensation of chronic disease were found to be independent predictive factors of 1-year mortality after surgery among rural older patients.

The higher mortality risk of the rural population compared with that of the urban population and the higher incidence of deaths during the first months after surgery revealed by our study are consistent with the results of other authors [9, 10]. These differences in rural–urban areas might be attributable to patient severity or variations in the quality of in-hospital or post-discharge care. There has been longstanding concern that rural populations might experience an increase in mortality compared with urban populations [8, 9] because of their reduced access to specialists. Other reasons for this finding of high mortality in rural older people could be due to differences in multimorbidity between urban and rural populations [12]; however, no differences were found in our sample. Furthermore, we could not find demographic, socioeconomic, social, or environmental factor differences in these aspects to explain the higher mortality in the rural population, as has been proposed previously [11, 13].

One possibility to consider is that rural older adults may have less monitoring and control of chronic diseases, among other inequities in access to health services such as rehabilitation and specialized consultations, which could explain the vulnerability in this population. Inequalities between urban and rural municipalities regarding follow-up health care services or rehabilitation services are common in the Andes Mountains [19]. Another reason, as previously emphasized, is that long travel distances might simply be an inconvenience for patients to adequately control their chronic medical conditions (e.g., hypertension, diabetes); thus, extended distances could result in treatment delays that increase patient mortality [8]. Further research is needed to clarify the role of distance and mortality in rural areas. In addition, the overall death occurrence of 32.5% observed in our study was similar to that in most previous extensive epidemiological studies [18]; the mortality rates following hip fractures worldwide vary but are generally high, as shown in our study [6]. Future research is warranted to identify other factors that might contribute to explaining the urban–rural mortality differences observed during the first years after hip fracture.

Our sample identified three statistically significant postoperative variables that predicted a greater mortality risk at 12 months following hip fracture surgery. According to previous studies [16, 19–27], these variables included anemia (a Hb level ≤ 9.0 g/dL during hospitalization), a postoperative blood transfusion requirement, and acute postoperative decompensation of chronic disease. Whether anemia is indeed associated with hip fracture mortality remains controversial [20]. Similar to our results, prior studies indicated that Hb levels might fluctuate in hip fracture due to various factors, including comorbidities such as chronic conditions, nutrition deficiencies, postoperative complications, the type of fracture, and in-hospital interventions [20]. Our study showed that rural patients had an increased rate of anemia on admission compared with their urban counterparts, and Hb levels decreased after surgical intervention, with a postoperative level ≤ 9.0 g/dL being another risk factor for mortality 1 year later. Thus, admission and postoperative anemia identify high-risk patients and contribute to higher mortality. However, further research is needed to identify adverse outcomes from anemia at different time points in rural patients with hip fractures. In our study, 65% of the rural patients were anemic on admission, 39% of whom received a transfusion. The requirement for transfusion following geriatric hip fracture has a significant association with morbidity and mortality [24]. Similar to other studies, patients who received a blood transfusion for hip fracture had other risk factors for mortality, including extracapsular fractures, admission and in-hospital anemia, and complications of comorbidities [24, 25].

The current study showed that medical complications during the postoperative period were associated with mortality 1 year later; these complications included COPD exacerbation, acute decompensation of heart failure, acute renal failure superimposed with preexisting chronic kidney failure, hyperosmolar state in diabetic patients, and hypertensive emergencies. All these complications have been reported to be associated with 1-year mortality after hip fracture decompensation [26, 27]. Recently, at least 44 prognostic factors of in-hospital complications after hip fracture surgery from 56 studies were identified, including dehydration, anemia, hypotension, heart rate variability, pressure risk, nutrition, and indwelling catheter use [28]. These complications should be prioritized in quality improvement efforts that target this patient population.

However, the lack of homogeneity in medical and surgical postoperative complications reported in those studies indicates that improving the reporting of complications in hip fracture trials with older populations is necessary. A standardized protocol for assessing and reporting complications should be developed and endorsed by researchers. Recently, one proposal regarding postoperative complications for hip fracture included 10 specific clinical variables that serve as quality metrics for the perioperative care of hip fracture repair patients to promote the possibility of unifying outcomes in hip fracture [29]. As suggested previously, it is now necessary that these postoperative complications be endorsed by professional organizations and, most importantly, by clinical investigators to provide the opportunity to compare results among future hip fracture mortality studies [30].

Another variable that emerged as a significant predictor of 1-year mortality was fracture type. Previously, the type of fracture was identified as an independent predictor of long-term mortality in patients with hip fractures, and extracapsular fractures, with the intertrochanteric type being more common, yield a worse prognosis, as shown in our study [31, 32]. To address the mechanism(s) by which intertrochanteric fractures lead to excess mortality compared to femoral neck fractures, future studies with hip fracture patients should include a comprehensive assessment of different aspects related to the severity of the fracture, including the degree of frailty, vitamin D status, and fall dynamics.

Unlike previous studies, our results show that the time from the fall to surgery and the time from admission to surgery were not significantly associated with 1-year mortality in rural older people. Previously, a delay in the time of surgery has been recognized as the most important factor related to mortality [19]. However, the association of surgical delay with increased mortality risk and complications is controversial [33]. We found that factors other than the time to surgery were related to higher mortality in rural areas. Previously, in the urban Colombian study mentioned above, it was hypothesized that the exacerbations and decompensations of pathologies could be due to the fracture or the prolonged prehospital delay (16). Similar to this study, the general mortality rates found in our study (20% at 30 days, 26% at 6 months, and 32% at 1 year postoperatively) were similar to the mortality rates reported by other studies [9, 16, 26], despite our mean surgical delay of 4 days.

The majority of variables found as significant predictors of mortality are included in the different predictive models proposed to stratify mortality risk [34]; however, these models were not developed for the rural population. Further research about comparisons between other predictive models proposed in this population is warranted. A simpler and faster tool to apply for older people who come from rural areas would permit the early estimation of the prognosis of hip fracture surgery and can help orthogeriatric teams in planning the perioperative care to avoid short- and long-term mortality [34].

Several strengths should be mentioned. One strength of this study is that it is the first study in Latin America to describe the differences in the 1-year mortality of rural/urban older patients with hip fractures. Additionally, the prospective design of this study helped us to see and evaluate the implications of significant pre- and postoperative complications on the risk of death in rural areas.

There are some limitations to this study that merit mention. First, our cohort was limited to one center, and extrapolation to a population outside this center must be made with caution. Second, information on prehospital care that might have been received was not available in this cohort of rural and urban patients. Thus, medical conditions and complications related to fractures are important avenues for future research to confirm the role of chronic conditions in the evolution of hip fracture. Third, the associated factors in the study are not causal, so more studies are required to confirm what was observed.

Compared with their urban counterparts, higher mortality in patients in rural areas could reflect disparities in health status or lifestyle, medical care for chronic conditions, differences in post-hip fracture health care, or combinations of several factors. Thus, older people with rural residency have been considered a structural determinant of becoming disabled [35]. Overall, our results provide additional knowledge useful for developing strategies to reduce the very high mortality after hip fracture in the Andes Mountains.

In conclusion, fragility hip fractures have higher mortality in rural older people than their urban counterparts at the 1-year follow-up. Several predictive factors were associated with this excess mortality, including a low hemoglobin level, transfusion requirement, fracture type, and acute postoperative decompensation of chronic disease. The high mortality in hip fracture patients remains a challenge both in rural and urban older individuals. The optimization of post-fracture treatment and care for chronic conditions could reduce the mortality of hip fractures in rural older individuals. However, the reasons for the excess mortality in rural areas should be explored as to whether mortality is a direct consequence of hip fracture, resulting from preexisting/comorbid medical conditions, or due to postoperative complications from these medical conditions or other socioeconomic factors.
